# The Use of CBD and Its Synthetic Analog HU308 in HIV-1-Infected Myeloid Cells

**DOI:** 10.3390/ph16081147

**Published:** 2023-08-12

**Authors:** Anastasia Williams, Pooja Khatkar, Heather Branscome, Yuriy Kim, James Erickson, Mohammad-Ali Jenabian, Cecilia T. Costiniuk, Fatah Kashanchi

**Affiliations:** 1 Laboratory of Molecular Virology, School of Systems Biology, George Mason University, Discovery Hall Room 182, 10900 University Blvd., Manassas, VA 20110, USA; 2 Department of Biological Sciences and CERMO-FC Research Center, University of Quebec in Montreal, Montreal, QC H2L 2C4, Canada; 3 Infectious Diseases and Immunity in Global Health Program, Research Institute of the McGill University Health Centre, Montreal, QC H4A 3J1, Canada; 4 Department of Medicine, Division of Infectious Diseases and Chronic Viral Illness Service, McGill University Health Centre, Montreal, QC H4A 3J1, Canada

**Keywords:** HIV-1, CBD, HAND, HU308, HU-308, cannabidiol, human immunodeficiency virus type 1, EVs, extracellular vesicles

## Abstract

Currently, there is no cure for human immunodeficiency virus type 1 (HIV-1) infection. However, combined antiretroviral therapy (cART) aids in viral latency and prevents the progression of HIV-1 infection into acquired immunodeficiency syndrome (AIDS). cART has extended many lives, but people living with HIV-1 (PLWH) face lifelong ailments such as HIV-associated neurocognitive disorders (HAND) that range from asymptomatic HAND to HIV-1-associated dementia. HAND has been attributed to chronic inflammation and low-level infection within the central nervous system (CNS) caused by proinflammatory cytokines and viral products. These molecules are shuttled into the CNS within extracellular vesicles (EVs), lipid bound nanoparticles, and are released from cells as a form of intercellular communication. This study investigates the impact of cannabidiol (CBD), as a promising and potential therapeutic for HAND patients, and a similar synthetic molecule, HU308, on the EVs released from HIV-1-infected myeloid cells as well as HIV-1-infected 3D neurospheres. The data shows that both CBD and HU308 decrease non-coding and coding viral RNA (TAR and *env*) as well as proinflammatory cytokines as *IL*-1β and *TNF*-α mRNA. This decrease in viral RNA occurs in in vitro differentiated primary macrophages, in EVs released from HIV-1-infected cells monocytes, and infected neurospheres. Furthermore, a 3D neurosphere model shows an overall decrease in proinflammatory mRNA with HU308. Finally, using a humanized mouse model of HIV-1 infection, plasma viral RNA was shown to significantly decrease with HU308 alone and was most effective in combination with cART, even when compared to the typical cART treatment. Overall, CBD or HU308 may be a viable option to decrease EV release and associated cytokines which would dampen the virus spread and may be used in effective treatment of HAND in combination with cART.

## 1. Introduction

Over 38 million cases of human immunodeficiency virus type 1 (HIV-1) were reported in 2021 [1,2,3]. Left untreated, HIV-1-infection advances into acquired immunodeficiency syndrome (AIDS) in which the depletion of CD4+ T-cells creates a weakened immune system that allows for opportunistic infections and may eventually lead to death [2,3,4]. Currently, there is no cure for HIV-1, but combined antiretroviral therapy (cART) extends the life of people living with HIV-1 (PLWH) by targeting multiple steps of the viral life cycle [5].

Although cART has extended lives of PLWH, potentially reminiscent to those free of infection, HIV-1 associated neurocognitive disorders (HAND) are still prevalent in approximately 50% of infected individuals [2,6,7]. HAND encompasses a variety of syndromes from asymptomatic to HIV-1-associated dementia [6,8]. HAND is attributed to chronic inflammation and low-level viral products within the central nervous system (CNS) that lead to neuronal injury and death [2,9,10]. Viral products persist in the CNS due to the lack of inhibitor for viral transcription which leads to low-level production of viral RNA [5,9,11,12]. Furthermore, the induction of transcription and lack of transcription latency is caused by multiple factors including stimulation by exosome shedding from uninfected cells [13,14]. Viral products are then released from the infected cells within extracellular vesicles (EVs) [15,16], causing both direct and indirect neuronal injury which contribute to the onset of HAND [17,18].

EVs are lipid bound nanoparticles that range in size from less than 50 nm to 5 µm [19,20]. EVs carry a variety of cargo including nucleic acids, proteins, chemokines, and kinases that are important for intercellular communication [4,19,20,21,22,23]. EVs can be separated by density via differential ultracentrifugation with varying speed that allows separation of different subpopulations, namely 2K, 10K, 100K, 167K short (167K(S)), and 167K long (167K(L)) each composed of different types of EVs, mainly large EVs (lEVs), microvesicles (MVs), exosomes, small EVs (sEVs), and exomeres, respectively [24,25,26,27,28,29,30]. The biogenesis of the EV subpopulations differ among various EVs and are not yet fully understood, but the primary methods of biogenesis involve budding directly from the plasma membrane (i.e., MVs) or via the endocytic pathway (i.e., multivesicular bodies for exosomes and others including amphisomes and autophagosomes) [24,26]. EVs have been shown to play a major role in the pathogenesis of a variety of viral infections including HIV-1 [11,13,23,31,32]. Specifically, EVs carrying HIV-1 viral products have been shown to elicit an immune response within recipient cells that increases susceptibility to infection and initiates the NF-κB pathway leading to the production of proinflammatory cytokines (*TNF*-α, *IL*-1β), which can lead to neuronal injury and death as seen with HAND [5,11,12,14,33,34].

Interestingly, cannabis use in PLWH is associated with a decrease in HAND [35,36]. This is thought to be due to the decrease of inflammation within the CNS from use of cannabinoids within the cannabis such as THC and CBD [35,37]. Previous studies have shown one of the major chemical components of marijuana, the phytocannabinoid cannabidiol (CBD), has the ability to decrease inflammation without the psychoactive effects associated with tetrahydrocannabinol (THC) [38,39,40,41,42,43]. Furthermore, CBD is FDA approved for epilepsy treatment [12,44]. Importantly, CBD has been shown to cross the blood–brain barrier (a layer of endothelial cells that regulates the exchange of molecules, ions, and cells between the blood and CNS) that prevents the entry of many drugs into the CNS (including components of cART) [12,45]. Furthermore, our lab has previously shown that CBD was found to not only decrease the viral cargo within EVs released from HIV-1-infected cells but to also reduce the number of EVs released from HIV-1-infected cells [4]. Thus, CBD has been proposed as a potential therapeutic for HAND in combination with cART.

Due to the anti-inflammatory effects of CBD observed previously, and the decrease in HIV-1 products, we believe that CBD and synthetic analogs such as HU308 could aid in the reduction of chronic inflammation by reducing the packaging of harmful material in EVs. However, this has yet to be fully developed, and this study aims to characterize the EVs released from HIV-1-infected monocytes after treatment with cannabidiol or its synthesized analog, HU308. EVs were separated by density centrifugation to create the subpopulations 2K, 10K, 100K, 167K(S), and 167K(L) and were analyzed for viral RNA and proinflammatory cytokine mRNA. Our data show that CBD or HU308 treatment reduced the amount of 100K EVs released as well as decreased the viral RNA within the 100K subpopulation. Similarly, both a primary macrophage model and a 3D neurosphere infection model showed CBD and HU308 treatment was associated with a decrease in viral RNA. Interestingly, it was with HU308 and cART in the neurosphere culture that showed a decrease in proinflammatory RNA. Furthermore, HU308 treatment in humanized mice infected with HIV-1 showed a decrease in HIV-1 RNA, while a combination of low cART with HU308 treatment showed significant decrease of viral RNA compared to the cART control.

## 2. Results

### 2.1. CBD Decreases the Classical Exosome EV Subpopulation

EVs released from virally infected cells have been shown to have a role in viral pathogenesis in a variety of infections including HIV-1 [23,31]. Even while on cART treatment, EVs released from virally infected cells still contain viral products due to the ongoing low-level viral transcription. These EVs interact with surrounding cells and induce proinflammatory cytokine cascades and prime the recipient cells for infection [14]. CBD has been shown to reduce inflammation in a variety of diseases (i.e., arthritis, osteoarthritis, epilepsy, Parkinson’s) and therefore is a promising therapeutic for HAND [38,39,40,46,47].

**Figure 1:**
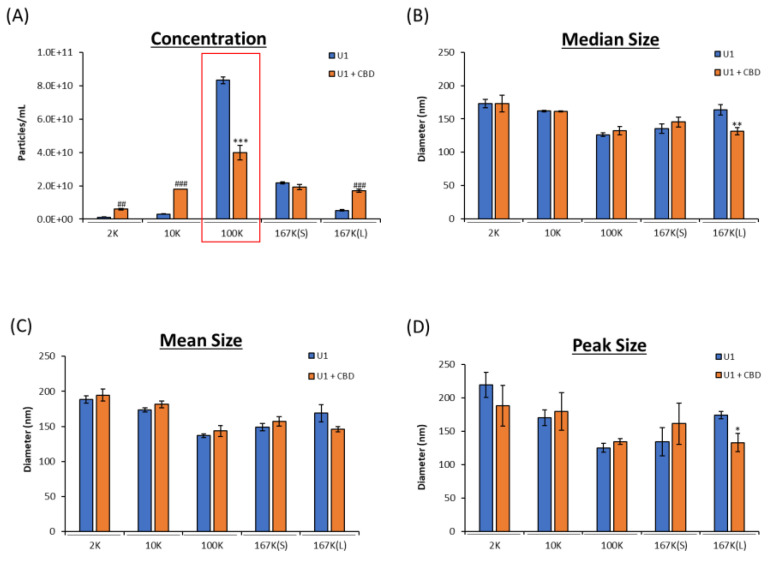
EV concentration and size released from HIV-1-infected monocytes with and without CBD treatment. U1 cells (1 × 10^6^ cells/mL) were treated with or without CBD (5 µM) for 3 days. At 72 h, cells were collected, and supernatant was spun at *g* × force 2K, 10K, 100K, 167K(S), and 167K(L). Concentration (**A**) and nanoparticle size (**B**–**D**) were measured through ZetaView analysis. Student’s *t*-test compared treated subpopulations to untreated. Red box indicates the subpopulation of interest. */#, *p* < 0.05; **/##, *p* < 0.01; ***/###, *p* < 0.001 where * represents a significant decrease, and # represents a significant increase compared to untreated. Error bars, S.D.

Our lab has previously shown that CBD (1–10 µM) reduces the concentration of EVs released from HIV-1-infected cells, while decreasing viral RNA and proteins both intracellularly and extracellularly [4]. This study provided the groundwork for dosing, in which we found 5 µM to be sufficient in providing a significant decrease in both EVs and viral products released from HIV-1-infected cells [4]. To explore this further, EV subpopulations (i.e., 2K, 10K, 100K, 167K(S), 167K(L)) were characterized. Here, U1 cells were treated with CBD (5 µM) daily for 3 days. After 72 h, supernatant was collected and processed using differential ultracentrifugation. The supernatant was then analyzed using ZetaView, and EV concentration and size were analyzed (Figure 1). From these results, CBD treatment was shown to result in a significant decrease within the 100K subpopulation (Figure 1A). Interestingly, the 2K, 10K, and 167K(L) had a slight increase in concentration after CBD treatment (Figure 1A), potentially pointing to specific regulation of exosome biogenesis via CBD. This change in biogenesis does not appear to affect size, as the only change in EV size was the 167K(L) subpopulation which had a slight decrease in size (Figure 1B–D). Collectively, these data indicate that CBD treatment leads to an overall decrease in EVs released from HIV-1-infected monocytes through the selective decrease in classical exosome subpopulation.

### 2.2. CBD Decreases Viral RNA and Proinflammatory Cytokine mRNA

**Figure 2:**
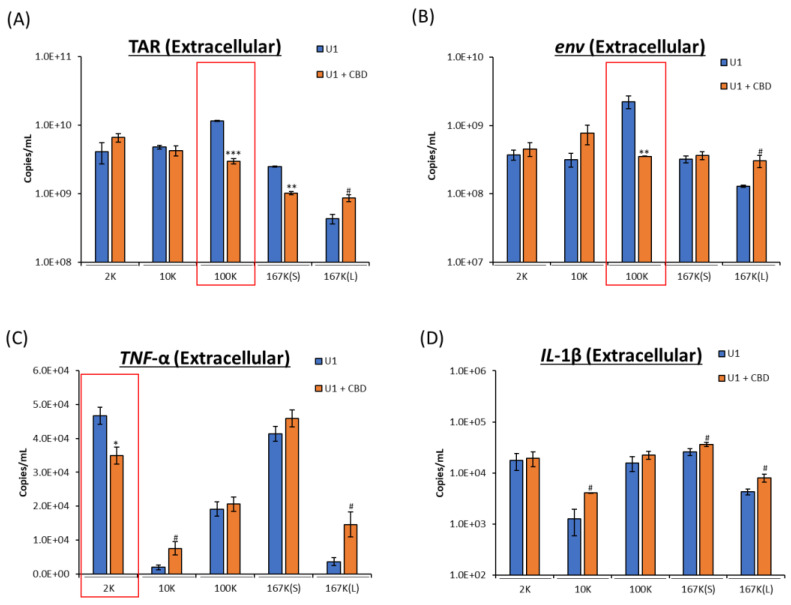
Levels of viral and proinflammatory cytokine RNA were assessed from EVs secreted from HIV-1-infected cells. U1 cells (1 × 10^6^ cells/mL) were treated with or without CBD (5 µM) for 3 days. At 72 h, cell pellets were collected, and EV subpopulations were isolated (2K, 10K, 100K, 167K(S), and 167K(L)). EV subpopulations were assessed for viral RNA (TAR and *env*) (**A**,**B**), as well as proinflammatory cytokine mRNA (*TNF*-α and *IL*-1β) (**C**,**D**). Subpopulations of interest are highlighted with red boxes. Student’s *t*-test compared treated subpopulations to corresponding untreated subpopulations. */#, *p* < 0.05; **/##, *p* < 0.01; ***/###, *p* < 0.001 (in which * represents a significant decrease, and # represents an increase compared to untreated). Error bars, S.D.

As discussed above, EVs released from infected cells contain viral RNA and proteins that promote pathogenesis. In our previous work, we showed that CBD decreases viral RNA and proteins found within EVs [4]. To determine the EV subpopulations impacted by CBD treatment, we analyzed the viral RNA in the 2K, 10K, 100K, 167K(S), 167K(L) subpopulations after treating the cells with CBD. Similar to EV concentration, CBD treatment caused a significant decrease in viral TAR and *env* RNA within the 100K subpopulation and a slight increase in the 167K(L) subpopulation (Figure 2A,B). As expected, there was an increase in the unprocessed viral spike protein gp160 with a reduction in the cleaved form of gp120 [4], which was found in the 167K(S) and 167K(L) subpopulations released from CBD-treated cells (Supplementary Figure S1).

Although viral products are known to be associated with neuronal damage, the major cause of neurotoxicity observed with HIV-1 infection is through the innate immune system, induced by the release of proinflammatory cytokines such as *TNF*-α and *IL*-1β [12]. CBD has been shown to decrease inflammation in a variety of diseases including neurological disorders, such as epilepsy and multiple sclerosis [41,48]. Specifically, CBD has been shown to attenuate the production of proinflammatory factors such as *IL*-1β and *TNF*-α to lessen the severity of neuronal inflammation within these disorders which can alleviate seizures and increase mobility, respectively [41,48].

Since HAND is characterized by the neuroinflammation, we assessed the level and impact of CBD on proinflammatory cytokines *IL*-1β and *TNF*-α through the levels of mRNA associated with EVs. EV associated mRNA has been shown to be translated by recipient cells, and there is further evidence that mRNA such as *IL*-1β can impose non-translational changes to the recipient cell as well (such as the upregulation of antiapoptotic gene expression in natural killer cells) [49,50,51,52]. Therefore, we attempted to determine which subpopulation(s) of EVs had proinflammatory cytokine mRNA and whether CBD treatment had any impact on the packaging of these populations. To ensure full length mRNA was analyzed, we synthesized the cDNA with an oligo(dT) primer (3′ extension) and investigated copy numbers with primers that were close to the cap (5′ end).

Results showed that both mRNAs were present in varying amounts in these fractions (Figure 2C,D). However, with CBD treatment, the decrease in proinflammatory cytokine mRNA *(IL*-1β and *TNF*-α) was not observed in the 100K subpopulation (compared to decrease viral products in the 100K fraction), and there was a decrease in the *TNF*-α mRNA levels within the 2K subpopulation (Figure 2C,D). This decrease may be from redistribution of cytokine mRNA between the EV subpopulations which showed altered levels in the 10K, 167K(L), and 167K(S), or the differences in EV subpopulations may be due to the increase in autophagy activation with CBD treatment shown previously [4] and its impact on EV regulation (Supplementary Figure S2). Taken together, CBD treatment shows a decrease in viral RNA transcripts TAR and *env* within the 100K subpopulation and decrease in proinflammatory cytokine mRNA *TNF*-α in the 2K subpopulation, likely indicating a package specific mechanism that is different for viral vs. cytokine RNA.

### 2.3. Analog of CBD (HU308) Similarly Reduces EV Subpopulations

**Figure 3:**
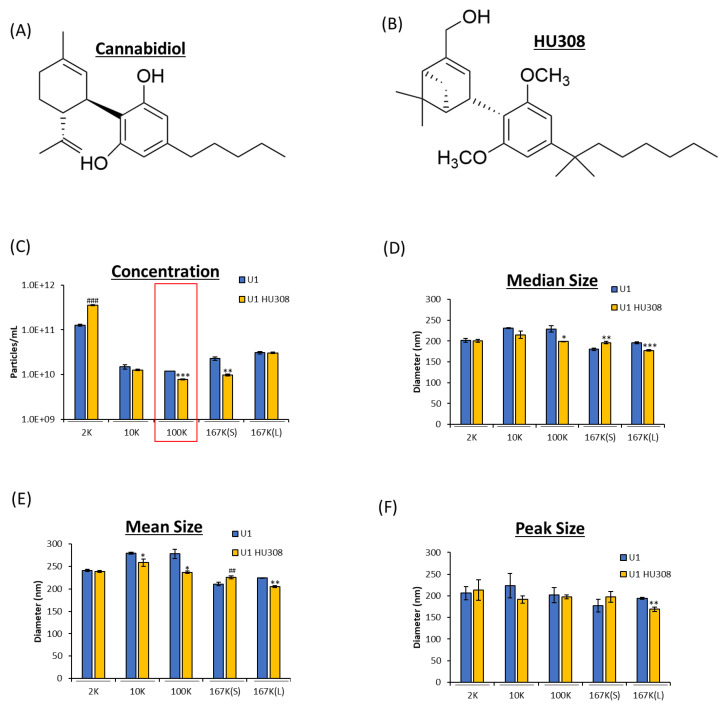
HU308 is a synthesized molecule, similar in structure to CBD, and has been proposed as an alternative anti-inflammatory. The molecular structures of CBD (**A**) and HU308 (**B**) is shown (biorender.com (accessed on 26, June 2023)). EV concentration (**C**) and size released from HIV-1-infected monocytes with and without HU308 treatment. U1 cells (1 × 10^6^ cells/mL) were treated with or without HU308 (5 µM) for 3 days. At 72 h, cells were collected, and supernatant was spun at *g* × force 2K, 10K, 100K, 167K(S), and 167K(L). Concentration (**C**) and nanoparticle size (**D**–**F**) were measured through ZetaView analysis. The red box indicates the subpopulation of interest. Student’s *t*-test compared treated subpopulations to untreated. */#, *p* < 0.05; **/##, *p* < 0.01; ***/###, *p* < 0.001; with * demonstrating a significant decrease and # a significant increase compared to untreated monocytes. Error bars, S.D.

The FDA has currently approved CBD in the medication Epidiolex^®^ for the treatment of seizures [53]. Epidiolex^®^ has been shown to be effective. However, CBD, with FDA approval, is isolated from the cannabis plant, and the purification process is expensive. It must go through validation and reproducibility in order to procure the correct dose at large quantities [54]. We therefore looked for a synthetic alternative with a structure similar to CBD’s that also had anti-inflammatory effects (i.e., *IL*-1β and *TNF*-α) and settled on HU308 [42]. HU308 is an analog to CBD and is a specific agonist for CB2 receptor [55], as opposed to CBD’s effect on GPR55, 5HT(1A), A2A, TRPV1-2 receptors, among others [56] (Figure 3A,B). CB2 is expressed mainly in the immune system [57], while the CBD receptors are within CNS and throughout the body (i.e., TRPV1 is found on neurons in epithelial cells [58,59]). These receptors are part of the G-coupled protein receptors and are pharmacologic targets of interest as they control inflammation as well as other sensations (i.e., pain) [37,60,61,62,63]. Thus, it is not surprising that HU308 has been shown to decrease peripheral and ocular inflammation [55,61].

Here, we aimed to investigate both CBD and HU308 treatment on HIV-1-infected cells, with HU308 showing no cell death at concentrations ranging from 0.1 to 100 µM during 3-day treatment of uninfected monocytes and T-cells (U937 and Jurkat, respectively) as well HIV-1-infected monocytes and T-cells (U1 and J1.1, respectively) (Supplementary Figure S3). Similar to CBD treatment, HU308 decreased EVs from HIV-1-infected cells released from the 100K but also within 167K(S) subpopulation (Figure 3C). HU308 treatment, however, also shows a slight decrease in EV size across subpopulations 10K, 100K, and 167K(L) while increasing 167K(S), indicating differences between CBD and HU308 treatments (Figure 3D,E). Collectively, these data indicate that HU308 may be a synthesized alternative to CBD in regulating EV release.

### 2.4. HU308 Reduces Both Viral and Proinflammatory RNAs

**Figure 4:**
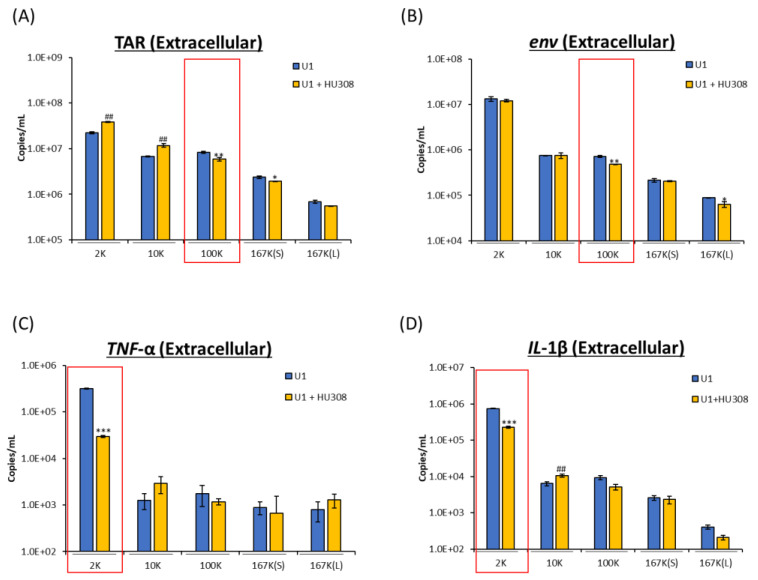
Levels of viral and proinflammatory cytokine RNA were assessed from EVs secreted from HIV-1-infected cells treated with HU308. U1 cells (1 × 10^6^ cells/mL) were treated with or without HU308 (5 µM) for 3 days. EV subpopulations were collected after differential ultracentrifugation spun at varying *g* × force, namely 2K, 10K, 100K, 167K(S), 167K(L). EV subpopulations were assessed for viral RNA TAR (**A**), *env* (**B**), and proinflammatory cytokine mRNA *TNF*-α (**C**) and *IL*-1β (**D**). Student’s *t*-test compared treated subpopulations to untreated. Red boxes indicate subpopulations of interest. */#, *p* < 0.05; **/##, *p* < 0.01; ***/###, *p* < 0.001; * depicts a significant decrease and # a significant increase compared to untreated. Error bars, S.D.

We next asked whether HU308 could regulate HIV-1 RNA levels as well as cytokine mRNA associated with the EVs released from HIV-1-infected cells. To determine the impacts of HU308 on HIV-1 infection and inflammation, a similar protocol was used as above. Similar to CBD, HU308 treatment reduced viral TAR and *env* RNA from the 100K EV subpopulation, as well as a slight decrease of TAR in the 167K(S) and *env* in the 167K(L) subpopulations (Figure 4A,B). Furthermore, we observed a decrease in *TNF*-α and *IL*-1β mRNA in the 2K EV subpopulation. This was seen with an increase in the 10K subpopulation (Figure 4B,C). Taken together, these data indicate that HU308 reduce viral RNA within the classical exosome subpopulation and proinflammatory cytokine mRNA (*TNF*-α and *IL*-1β) within the 2K subpopulation, similar to CBD, indicating a similar package specific response to the drug treatment.

### 2.5. Decrease in Viral RNA in Primary Macrophages Treated with HU308 or CBD

During the initial HIV-1 infection, there is an increase in mobility of activated peripheral blood monocytes to the brain, and it is the microglial (the brain’s resident macrophage population) in which the viral reservoir replicates and contributes to the neuroinflammation observed in HAND [2,64]. We next utilized primary macrophages for infection with dual tropic virus (89.6) and treatment with HU308. Previously, we had shown that CBD decreases viral RNA in primary cells. However, in this study, we aimed to explore the impact of HU308 as well as the combination of these cannabinoids with cART [4].

**Figure 5:**
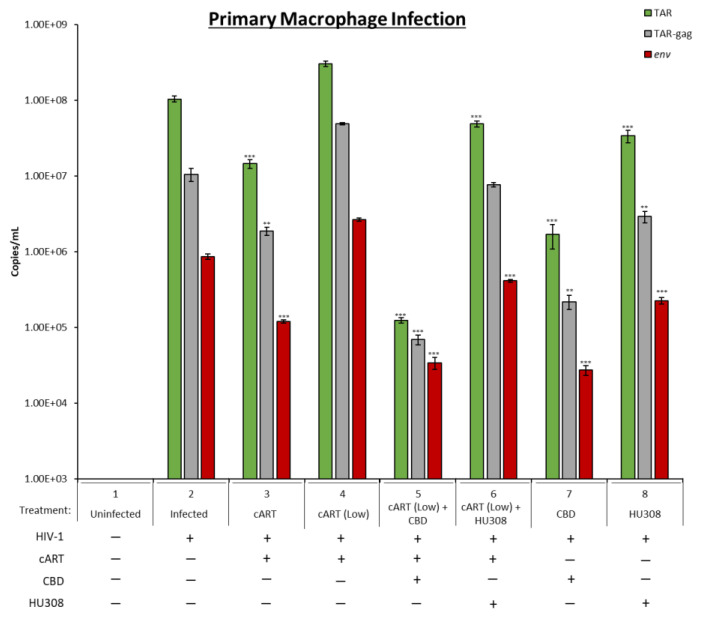
Levels of viral RNA were assessed from HIV-1-infected primary macrophages. Primary PBMCs were purchased (Precision For Medicine, Frederick, MD, USA) and cultured in vitro with PMA/PHA every other day for 10 days. The adherent macrophages were grown for 8 more days with fresh media and PMA treatment given every other day. On day 8, the cells were infected with HIV-1 89.6 strain (MOI:10), and the following day, treatment with cART (Lamivudine, Tenofovir, Emtricitabine, Indinavir; 10 µM, or low concentration of 1 µM each), CBD (5 µM), HU308 (5 µM), or combination was utilized every 3rd day for a total of 3 treatments. On the 2nd treatment, fresh media was added. On day 10, cell pellets were collected, and RNA was isolated and assessed for TAR, TAR-*gag*, and *env*. Student’s *t*-test compared treated subpopulations to untreated. *, *p* < 0.05; **, *p* < 0.01; ***, *p* < 0.001. Error bars, S.D.

Here, peripheral blood mononuclear cells (PBMCs) were differentiated into macrophages, infected with HIV-1 dual tropic strain and then treated with CBD, HU308, cART, or combination every 3 days for 10 days. The cells were then collected on day 10, and the RNA was isolated and assessed for presence of viral TAR, TAR-*gag,* and *env.* HIV-1 non-coding TAR represents short transcripts; TAR-*gag* represents transcription into *gag* open reading frame; and *env* is representative of full-length transcription observed in infections. When cells were infected with HIV-1 89.6, there was an increase in all three transcripts after 10 days. Treatment with optimal concentration of cART (10 µM each) showed a significant decrease in all three RNA transcripts (Figure 5; lane 3). We then treated cells with lower levels of cART (1 µM of each anti-viral compound) and observed no drop in HIV-1 transcription. However, when a combination of low cART and CBD was added to cells, there was a significant decrease in all transcripts (lane 5) compared to CBD alone (lane 7). Finally, when low cART and HU308 were added to infected cells, there were no significant changes in RNA levels compared to HU308 alone (lane 6 vs. 8). Taken together, these data indicate that CBD along with low cART have a more pronounced effect on HIV-1 RNA levels in primary macrophage infection compared to HU308 treatment.

### 2.6. Effect of HU308 and CBD in a 3D Mini-Brain Infection Model

**Figure 6:**
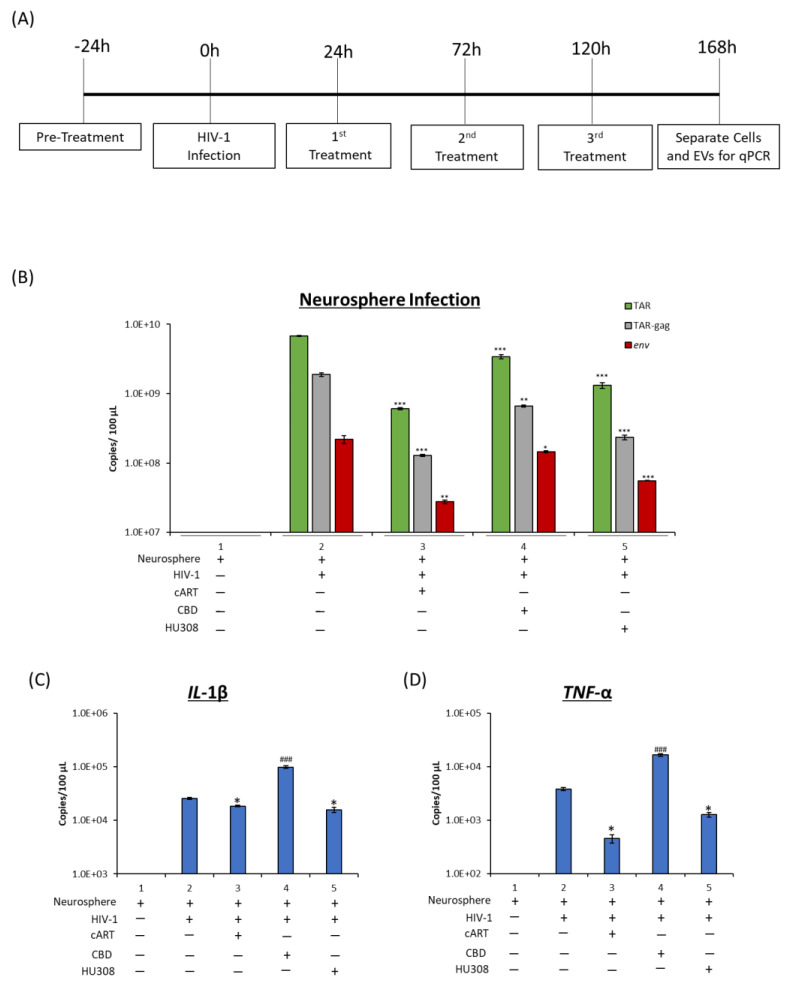
Levels of viral and proinflammatory RNA were assessed from neurospheres. Neurospheres were pre-treated with cART (Tenofovir, Emtricitabine, Lamivudine, and Indinavir; 10 μM), CBD (10 µM), or HU308 (10 µM) for 24 h prior to HIV-1 infection and treated again 24 h post-infection, then every 48 h for 7 days (**A**). RNA was isolated and assessed for levels of viral TAR, TAR-*gag*, and *env* RNA (**B**) as well as *IL*-1β (**C**) and *TNF*-α (**D**). Student’s *t*-test compared treated samples to untreated samples. */#, *p* < 0.05; **/##, *p* < 0.01; ***/###, *p* < 0.001 * represents a significant decrease, and # represents a significant increase compared to untreated, HIV-1-infected neurosphere. Error bars, S.D.

HAND is a comorbidity due to the low-level viral transcription and inflammation within the CNS. We therefore utilized a 3D infection model to score for the effects of HU308 and CBD. The 3D neurospheres used here have previously been shown to be composed of neuronal progenitor cells, neurons, astrocytes, and microglia-like cells and were shown to be a suitable model for HIV-1 infection and cART treatment [65]. In this study, neurospheres were pretreated with cART, CBD, or HU308 for 24 h prior to infection and then treated every 48 h for 7 days (Figure 6A). At 168 h post-infection, neurospheres were pelleted, and cells were collected for viral and cytokine RNA analysis. There was a decrease in intracellular HIV-1 RNA levels post cART (Figure 6B; lane 3) and a drop in viral RNA with CBD or HU308 alone (lane 4 and 5).

The neuronal death and injury associated with HAND is mainly caused by viral products and proinflammatory cytokines [2]. To better understand the potential decrease in proinflammatory cytokine RNA levels, infected neurospheres were treated with CBD and HU308 and assayed for viral RNA but also for proinflammatory cytokine mRNA in these cultures. We observed the presence of both cytokine RNAs in all infected neurospheres. As expected, with cART treatment, there was a decrease in *IL*-1β mRNA levels in these cultures (Figure 6C; lane 3). This trend was also seen post HU308 treatment (lane 5). Interestingly, there was an increase in *IL*-1β levels post-CBD treatments (lane 4). We also assayed for presence of *TNF*-α and again observed a similar trend in which there was a decrease in levels with cART or HU308 treatment and an increase with CBD (Figure 6D). Collectively, these data implicate that in a multi-cellular infection model (3D), CBD and HU308 can decrease viral RNA levels, and that HU308 can reduce *IL*-1β and *TNF*-α proinflammatory cytokine mRNA levels.

### 2.7. HU308 Treatment Reduces Viral RNA in Humanized Mice

As treatment with HU308 decreased viral RNA but and proinflammatory cytokine RNA in both monocytes and 3D neurospheres, we next asked if the decrease in viral RNA transcripts observed with HU308 treatment would be reproducible in a multiorgan system. To explore the reduction of viral transcription, we used the NOD.Cg-*Prkdc^scid^ IL2rg^tm1Wjl^*/SzJ (NSG) humanized mouse model infected with dual-tropic HIV-1 89.6 strain which we have previously used for HIV-1-infection model [66]. Mice (*n* = 18) were divided randomly into 2 groups, uninfected (*n* = 3) or HIV-1-infected (*n* = 15), where the mice were infected with HIV-1 89.6 dual-tropic strain and further divided into 5 treatment groups (*n* = 3): untreated, cART treated (at optimal levels), cART treated (low levels), HU308, or a combination of HU308 and cART (low levels). Low levels of cART were used in combination with HU308 to display the potential anti-viral effects of HU308. Our 1 mg/kg HU308 three time weekly intraperitoneal (IP) injection was similar to the twice weekly injection (1 mg/kg) which has been shown to be a safe and effective dose in a mouse model for the protection of ovariectomy-induced bone loss [67]. Furthermore, HU308 (IP 2 mg/kg) has been shown to be successful in the prevention of myocardial infarction damage by reducing inflammatory markers such as *IL*-1β and *TNF*-α [68]. Mice were treated every 48 h for 1 week, and at day 7, the plasma was collected for downstream RT-qPCR analysis.

**Figure 7:**
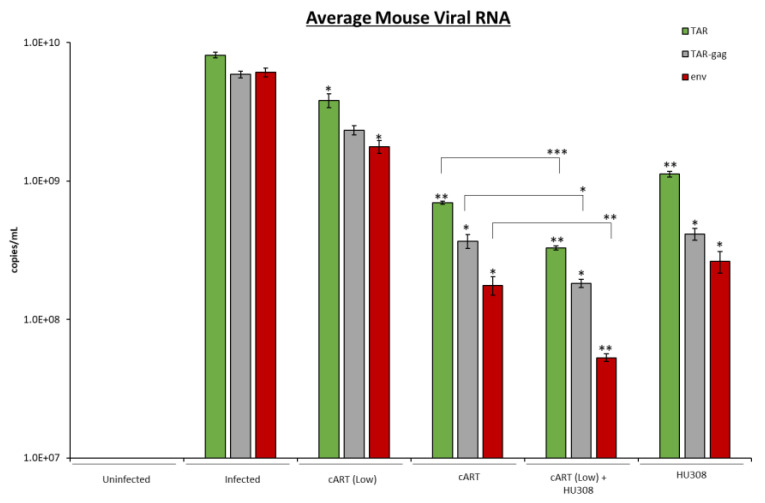
Levels of viral and proinflammatory RNA were assessed from NOD.CG-*Prkdc^dcid^ IL2rg^tm1Wjl^*/*SzJ* (NSG) humanized mice. NSG mice (*n* = 18) were infected with dual-tropic 89.6 strain as described in materials and methods and were treated with cART (emtricitabine 210 mg/kg; tenofovir 20 mg/kg; ritonavir 60 mg/kg; maraviroc 60 mg/kg) at cART low (1/10 cART), HU308 (1 mg/kg), or a combination of HU308 and cART low every 48 h over the course of a week. Plasma was collected, and RNA was assessed for viral TAR, TAR-*gag*, and *env* (shown as average). Student’s *t*-test compared treated samples to untreated samples, and cART low in combination of HU308 to cART high. *, *p* < 0.05; **, *p* < 0.01; ***, *p* < 0.001. Error bars, S.D.

We observed an attenuation in viral RNA transcription across all three mice treated with low levels of cART in combination with HU308 compared to the untreated, infected mice (Figure 7; lane 4 compared to 1). A similar trend was seen with HU308 treatment alone (lane 5). However, the decrease in viral RNA observed with the combination of HU308 and cART was statistically significant even when compared to the mice treated with the optimal amount of cART (lanes 4 and 3). Taken together, these data indicate that HU308 alone reduces HIV-1 viral replication in a humanized mouse model, and that HU308 in combination with cART has a greater potency in reducing viral replication than either HU308 or cART alone.

## 3. Discussion

In this manuscript, our principal aim was to characterize the extracellular vesicles released from HIV-1-infected monocytes treated with CBD and to compare effects of CBD to its synthesized analog HU308. CBD treatment and its effects on inflammation have been assessed previously; however, the impact of CBD and HU308 on EV regulation has not been fully studied [69,70].

CBD treatment significantly reduced the 100K EV subpopulation, while slightly increasing the 2K, 10K, and 167K(L) subpopulations. We previously showed that there was an overall decrease in EVs released from HIV-1-infected cells post-CBD treatment [4]. Therefore, lowering the 100K subpopulation (predominately exosomes) is consistent with previous observation as well as in other cancer lines [4,71]. Furthermore, CBD significantly decreased the viral TAR and *env* RNA within the 100K subpopulation. This again is consistent with the previously observed decrease in viral RNA released from HIV-1-infected monocytes treated with CBD [3]. Furthermore, there was an increase in unprocessed gp160 in 167K(S) and 167K(L) subpopulations which points to the presence of unprocessed envelope in both small exosome and exomere subpopulations.

Interestingly, CBD treatment reduced the proinflammatory cytokine mRNA *TNF*-α within the 2K subpopulation. However, there was a redistribution into 10K and 167K(L), and to some extent the 167K(S) subpopulation, indicating that biogenesis of 2K may be related to 10K and 167K(L) EVs. Alternatively, the 167K(L) has a lipid raft population which may hold mRNAs. Of note, the 2K subpopulation displayed an increase in p62 (Supplementary Figure S1), an indicator of autophagy and autophagosomes [72], which has been shown to play an important role in the reduction of proinflammatory cytokines such as *TNF-α* and *IL*-1β [73]. This decrease in *TNF*-α RNA in addition to a decrease in the 100K subpopulation (the classical exosome) may be from increased autophagy observed previously [3], as well as the decrease in clathrin-dependent endocytosis from decreased Hsc70 levels (Supplementary Figure S2) [74,75]. This may also point towards overall lowering the inflammation post-CBD treatment, which correlates with a drop in the 100K subpopulation, as even though the cytokine mRNA did not decrease the number of EVs, the mRNA decreased, resulting in total RNA decrease.

Isolating CBD can be tedious and expensive, so its synthesized analog, HU308, was included in this study. We found that HU308 did not cause cell death in uninfected nor infected monocytes and T-cells with treatment ranging from (0.1–100 µM), consistent with other publications in which HU308 has been shown previously to be safe up to 50 µM in primary murine macrophages [67,76]. Overall, HU308 had similar results to those of CBD. Specifically, we observed that HU308 treatment had a reduced number of 100K EVs released from HIV-1-infected monocytes, and that the 100K subpopulation had reduction in viral RNA TAR and *env.* Furthermore, when treated with HU308, there was a reduction of *IL*-1β and *TNF*-α in the 2K subpopulation.

The overall trend in decrease in viral RNA was observed in a primary macrophage model in both cannabinoids. However, to better understand the complexity of HAND, a 3D neurosphere model was used, and again, CBD and HU308 showed overall similar effects of decrease in viral RNA. Specifically, there was a decrease in viral TAR, TAR-*gag,* and *env*. Furthermore, there was also a reduction in *TNF*-α and *IL*-1β with HU308 treatment. Interestingly, we also performed similar infections for Human T-lymphotropic virus type-1 (HTLV-1) in the 3D neurosphere models and found that CBD and HU308 had both anti-viral and anti-inflammatory roles (Supplementary Figure S4). This is not the first study to show anti-viral effects with cannabinoids, as CBD has been shown to display anti-viral effects with Hepatitis C and Kaposi’s sarcoma-associated herpesvirus [77,78,79]. However, it is interesting that HU308 appears to show a more pronounced decrease in all HTLV-1 transcripts *tax*, *hbz,* and *env* (Supplementary Figure S4). Taken together, these data suggest that CBD and HU308 may reduce neuroinflammation by reducing the viral and proinflammatory transcripts through autophagy or mechanisms not yet fully understood.

To assess this further, we used a humanized mouse model of infection and found that HU308 was able to reduce viral TAR, TAR-*gag*, and *env* RNA. Interestingly, we observed the most effective treatment was a combination of HU308 with a low dose of cART. This treatment combination brought viral RNA levels down below those of even the optimal dose of cART. Overall, CBD or HU308 treatment showed a decrease in viral RNA and changed the characteristics of EV populations in regards to concentration and cargo (Table 1). Therefore, the known anti-inflammatory attributes of these cannabinoids may be based on a few different factors: the selective targeting in cargo observed by the decrease in *IL*-1β, *TNF*-α in the 2K subpopulation and decrease in viral RNA in the 100K subpopulation, the overall decrease of EVs released in the 100K subpopulation, and the decrease in overall intracellular products observed with the decrease in intracellular viral RNA, *TNF*-α, or through a combination. Taken together, CBD or HU308 in combination with cART may decrease neuronal inflammation seen in HAND by decreasing viral cargo and decreasing proinflammatory cytokine mRNA released from HIV-1-infected cells. Further testing should be done to consider these drugs as part of the cART regime.

Moving forward we aim to explore the mechanisms of each of these drugs in detail. For example, each of these drugs target different receptors, where HU308 mainly targets CB2, and CBD targets a variety of receptors including GPR55 and TRPV1. It is important to note that these receptors activate different pathways that impact inflammation [55,56]. There is also potential for studies outside of HAND pathology including chronic cancer inflammation which is associated with pain. It has been shown that to reduce the pain associated with chronic inflammation, both CB2 and TRPV1 are beneficial. Thus, targeting both receptors should be considered in future studies [60,80]. Furthermore, longevity studies should be done to determine effects of long-term drug use with CBD or HU308 in combination with cART, especially in regards to latency.

## 4. Materials and Methods

### 4.1. Cells and Reagents

HIV-1-infected (U1) monocytes provided by the National Institutes of Health’s (NIH) AIDS Reagent program were cultured in RPMI 1640 media containing 10% EV-depleted fetal bovine serum (FBS), 1% penicillin/streptomycin, and 1% L-glutamine (Quality Biological, Gaithersburg, MD, USA). Cells were incubated at 37 °C in 5% CO_2_. U1 cells were treated with Cannabidiol (CBD; 2-[1R-3-methyl-6R-(1-methylethenyl)-2-cyclohexen-1-yl]-5-pentyl-1,3-benzenediol; CAT: 90080, Cayman Chemical, Ann Arbor, MI, USA; 5 µM) or HU308 (((1S,4S,5S)-4-(2,6-dimethoxy-4-(2-methyloctan-2-yl)phenyl)-6,6-dimethylbicyclo [3.1.1]hept-2-en-2-yl)methanol; Tetrabiopharma and Targetedbioscience); 5 µM) for 3 days.

### 4.2. Primary Cells

A set of healthy, primary PBMCs was purchased (Precision For Medicine, Frederick, MD, USA) and cultured in vitro. The cells were placed in fresh complete RPMI overnight, and Phorbol 12-Myristate 13-Acetate (PMA; CAT: 16561-29-8, Cayman Chemical) and phytohaemagglutinin (PHA) were added the next morning. PMA/PHA was added every other day, and media were added throughout 10 days. On day 10, T-cells cells in suspension were removed and in vitro differentiated. Primary macrophages were allowed to grow with fresh media and PMA treatments for 8 more days. Macrophages were infected with HIV-1 89.6 strain (MOI:10). The next day, the infected cells were treated with cART at high levels (10 µM; Lamivudine, Tenofovir, Emtricitabine, Indinavir), low level cART (1 µM), and with or without CBD (5 µM) or HU308 (5 µM). This treatment was added every 3 days for a total of 3 treatments, and on the 2nd treatment, fresh media were added. Macrophages were collected on day 10 and processed for RNA analysis.

### 4.3. Serum EV-Depleted Medium

FBS was depleted of EVs as previously described [81], where FBS was at ultracentrifugation 100,000× *g* for 90 min in a Ti70 rotor (Beckman Coulter, Indianapolis, IN, USA). The EV-depleted supernatant was then carefully collected and stored at −20 °C for future use.

### 4.4. ZetaView Nanoparticle Tracking Analysis (NTA)

ZetaView Z-NTA (Particle Metrix, Inning, Ammersee, Germany) with software (ZetaView 8.04.02, Particle Metrix) was used to preform NTA. Machine calibration and user settings were previously described and performed in accordance to the manufacturer’s protocols [11,81]. The ZetaView software was used to analyze the mean size (diameter; nm) and the sample concentration (EV number) after the measurement from the 11 measured positions and removal of outliers. The measurement data provided from ZetaView were then inserted into Microsoft^®^ Excel^®^ to calculate averages and standard deviation from the technical triplicates.

### 4.5. EV Isolation via Differential Ultracentrifugation

HIV-1-infected monocytes (U1 cells) were grown in T75 flasks and expanded up to 25 mL of culture volume. The cells were treated with PBS or CBD (5 µM) daily for three days. The cells were then separated from the supernatant via low-speed centrifugation (300–400× *g* for 10 min). Supernatants were then transferred into ultracentrifugation tubes, and the tubes were placed in Ti70 (Beckman, Brea, CA, USA) rotor and spun at 2000× *g* (2K) at 4 °C for 45 min. Next, the supernatant was transferred to new tubes and further centrifuged at 10,000× *g* (10K) for 45 min, while the pellets were resuspended with PBS (150 µL per tube) and transferred to fresh 1.5 mL tubes. After the 10K spin, the supernatant was again separated into new tubes and spun at 100,000× *g* (100K) for 90 min, and the pellets were resuspended and transferred as described above. This process of supernatant removal and pellet resuspension was repeated, and the supernatant was spun down at 167,000× *g* for 4 h (h; 167K(S)), the pellet was collected, and the final spin was done at 167,000× *g* for 16 h (167K(L)). The final supernatant was discarded, and the 167K(L) pellet was resuspended in PBS. Isolated EVs were stored at −20 °C for future assays.

### 4.6. RNA Isolation, cDNA Synthesis, and Quantitative Real-Time PCR (RT-qPCR)

Total RNA isolation of cells and EVs were prepared as follows. Cells were harvested, washed once in 1× PBS free of calcium and magnesium, and resuspended in 50 µL of 1× PBS free of calcium and magnesium. EVs were isolated via differential ultracentrifugation as described above. Trizol Reagent (Invitrogen, Carlsbad, CA, USA) was used to isolate total RNA from cell pellets and isolated EVs according to the manufacturer’s protocol. GoScript Reverse Transcription Systems (Promega, Madison, WI, USA) was used to generate cDNA with Envelope Reverse: (5′-TGG GAT AAG GGT CTG AAA CG-3′), TAR Reverse: (5′-CAA CAG ACG GGC ACA CAC TAC-3′), and oligo(dT) reverse primer (Promega) with a GoScript kit (Promega). These were compared to quantitative standards that were created from serial dilutions of a CEM T-cell line that contained a single copy of HIV-1 LAV provirus per cell (8E5cells).

For viral RNA, cDNA samples (2 µL) or standards (2 µL) were plated in a master mix (18 µL) composed of IQ Supermix (Bio-Rad, Hercules, CA, USA), TAR Forward Primer (5′-GGT CTC TCT GGT TAG ACC AGA TCT G-3′), TAR Reverse Primer (5′-CAA CAG ACG GGC ACA CAC TAC-3′), or (viral *env* forward primer (5′-GGC AAG TCT GTG GAA TTG G-3′) and *env* reverse (5′-TGG GAT AAG GGT CTG AAA CG-3′) were used to measure full length viral genome) and TAR Probe (5′56-FAM-AG CCT CAA TAA AGC TTG CCT TGA GTG CTT C-36-TAMSp-3′). The BioRad CFX96 Real Time System was used for RT-qPCR with the following conditions: a single 2 min cycle at 95 °C followed by 41 cycles with 15 s at 95 °C and 40 s at 60 °C.

For all other RNA, cDNA samples or standards (2 µL) were plated with a master mix (18 µL) of SYBR Green (Bio-Rad) that included corresponding primers below and set to the following cycles.

*TNF*-α: forward primer (5′-CCC GAG TGA CAA GCC TGT AG-3′), reverse primer (5′-GAT GGC AGA GAG GAG GTT GAC-3′). The conditions were a single 50 °C 2 min, a 95 °C cycle for 2 min, then 41 cycles of 95 °C for 15 s, 57.3 °C for 30 s, and 72 °C for 40 s.

*IL*-1β: forward primer (5′-AGA TGA TAA GCC CAC TCT ACA G-3′), reverse primer (5′-ACA TTC AGC ACA GGA CTC TC-3′). Which were later verified with primers closer to the 5′ end: *IL*-1β forward (5′-TCA GCC AAT CTT CAT TGC TC-3′ and reverse (5′-AAC AAG TCA TCC TTG CC-3′). The BioRad CFX96 Real Time System conditions were set at 50 °C for 2 min, 95 °C for 2 min, and a 41 cycle of 95 °C for 15 s, 54 °C for 40 s, and 72 °C for 40 s.

*IL*-10: forward (5′-GCA AAA CCA AAC CAC AAG ACA GA-3′) and reverse (5′-TCT CGA AGC ATG TTA GGC AGG-3′). The conditions were as follows, the initial 50 °C for 2 min, then a single 95 °C cycle for 2 min, 41 cycles of 95 °C for 15 s, 59.0 °C for 30 s, and 72 °C for 40 s.

Cellular *GAPDH*: forward primer (5′-GAA GGT GAA GGT CGG AGT CAA C-3′), reverse primer (5′-CAG AGT TAA AAG CAG CCC TGG T-3′), and conditions were set at 50 °C for 2 min, 95 °C for 2 min, 41 cycles of 95 °C for 15 s and 57.5 °C for 30 s.

Each reaction was performed in triplicate, and quantification was determined through the comparison of cycle threshold (Ct) values to the 8E5 standard curve in the BioRad CFX Manager Software. The analysis of generated raw data was performed in Microsoft^®^ Excel^®^.

### 4.7. Preparation of Whole Cell Extracts

HIV-1-infected monocytes (U1) cell pellets were collected, washed with 1× PBS, and resuspended in 50 µL of lysis buffer [50 mM Tris-HCl (pH 7.5), 120 mM NaCl, 5 mM EDTA, 0.5% Nonidet P-40, 50 mM NaF, 0.2 mM Na_3_VO_4_, 1 mM DTT, and 1 complete protease inhibitor cocktail tablet/50 mL (Roche Applied Science, Penzberg, Germany)]. The resuspended cells were then incubated for 20 min on ice with vortexing every 4 min. After the 20 min incubation, the cell debris were centrifuged at 10,000× *g* for 10 min. A Bradford protein assay was then performed in accordance of the manufacturer’s protocol (Bio-Rad) to determine protein concentrations of each cell lysate.

### 4.8. Western Blot

Laemmli buffer was added to isolated EV pellets or to sample cell lysates (15 µg) and was then heated for 3 min at 95 °C. Next, 10 µL of cell lysate sample and EV sample were then loaded onto a 4–20% Tris/glycine gel (Invitrogen). The gels were run at 100 V and then transferred onto Immobilon PVDF membranes (MilliporeSigma, Burlington, MA, USA) at 50 mA overnight. The membranes were blocked for 2 h at 4 °C with 5% milk in PBS with 0.1% Tween-20 (PBS-T). The gels were then incubated in PBS-T with the appropriate primary antibody overnight at 4 °C: α-gp120 (CAT: 522; NIH AIDS Reagent Program), α-GAPDH (CAT: 47724; Santa Cruz Biotechnology, Paso Robles, CA, USA), α-CD63 (CAT: EXOAB-CD63A-1; Systems Biosciences, Palo Alto, CA, USA), α-catalase (CAT: 724810; Novus Biologicals, Centennial, CO, USA), α-Phosphor-Cofilin (Ser 3) (CAT: 3311; Cell Signaling Technology), α-Rab7 (B-3) (CAT: 376362; Santa Cruz Biotechnology), α-HSP90 α/β (F-8) (CAT: 13119; Santa Cruz Biotechnology), α-Hsc70 HRP (B-6) (CAT: 7298; Santa Cruz Biotechnology), α-coronin-1A (CAT: NB110-58867; Novus Biologicals). Membranes were washed with PBS-T and then incubated at 4 °C for 2 h with the indicated HRP-conjugated secondary antibody. Clarity Western ECL Substrate (Bio-Rad) was used with the Molecular Imager ChemiDoc Touch System (Bio-Rad) to view HRP luminescence.

### 4.9. Cell Viability Assay

Cells (uninfected monocytes (U937), HIV-1-infected monocytes (U1), uninfected T-cells (Jurkat), HIV-1-infected T-cells (J1.1)) cultured in RPMI with 10% exosome-free FBS were plated at 5 × 10^4^ cells per well in 96-well plates. Cells were treated with HU308 at varying concentrations (0.1, 1, 5, 10, 100 µM) every 24 h for 3 days. A Cell Titer-Glo assay was used to measure cellular ATP activity according to the manufacturer’s protocol (Reaction Biology, Malvern, PA, USA). Briefly, 100 µL of Cell Titer-Glo was added to each well and then mixed for 2 min (in dark conditions) and left in room temperature undisturbed for 10 min. The readings were taken with GloMax Explorer (Promega) and analyzed in Microsoft^®^ Excel^®^.

### 4.10. Neurosphere 3D Model

Using STEMdiff™ Neural Progenitor Medium (STEMCELL Technologies™; Vancouver, BC, Canada), Neural Progenitor Cells (NPCs; CAT: ACS-5003; ATCC. Manassas, VA, USA) were grown and used to generate 3D neurospheres (1 × 10^5^ cells per well) as described before [65]. Briefly, 3D neurospheres were differentiated with DMEM:F12 (CAT: 30-2006; ATCC) with Neural Progenitor Cell Dopaminergic Differentiation Kit (CAT: ACS-3004; ATCC) and incubated for 14 days. Neurospheres were infected with 10 µL of Infectin™ (Virongy, LLC; Manassas, VA, USA) and HIV-1 89.6 dual-tropic strain (MOI:10) and were incubated for 48 h in a total volume of 200 µL. Neurospheres were treated with 1× PBS ± 10 µM cART cocktail (Lamivudine, Tenofovir, Emtricitabine, Indinavir) ± CBD (10 µM), ±HU308 (10 µM) every other day for 7 days. At day 7, RNA was isolated from neurospheres for downstream RT-qPCR analysis.

### 4.11. Humanized Mice

Since there is considerable interest in the use of selective CB_2_ receptor agonists (i.e., HU308, which are devoid of psychoactive properties of CB_1_ agonists), we utilized humanized mice to test its effect on HIV-1 replication. All mice used in this study were maintained within the National Center for Biodefense and Infectious Disease’s breeding colony (George Mason University, Manassas, VA, USA). All experiments were carried out in bio-safety level 3 (BSL-3) facilities and in accordance with the Guide for the Care and Use of Laboratory Animals (Committee on Care and Use of Laboratory Animals of The Institute of Laboratory Animal Resources, National Research Council, National Institutes of Health Publication 86-23, revised 1996). NOD.Cg-*Rag1^tm1Mom^ Il2rg^tm1Wjl^*/SzJ mice were obtained from The Jackson Laboratory (007799, Bar Harbor, ME, USA) and were humanized [66]. Briefly, five groups of three neonatal animals were sub-lethally irradiated. The next day, mice were intraperitoneally injected (100 µL) with ~5 × 10^5^ human cord blood-derived CD34^+^ hematopoietic stem cells (Lonza, Walkersville, MD, USA). At ~3 months post-engraftment, animals were subcutaneously infected with the dual-tropic HIV-1 89.6 (100 µL, 20 ng of p24/µL). Mice receiving cART (emtricitabine 210 mg/kg, tenofovir 20 mg/kg, ritonavir 60 mg/kg; maraviroc 60 mg/kg), HU308 (1 mg/kg) [67,68], or a combination of HU308 and cART were injected intraperitoneally three times a week prior to blood isolation, and PCR. HU308 was obtained from and dissolved in anhydrous ethanol at 20 mg/mL prior to preparation in 1:1:18 solution of HU308: Kolliphor EL: and saline stored at 4 °C.

### 4.12. Statistics

All analysis was performed through Microsoft^®^ Excel^®^ software 2019. Data were analyzed as independent two-sample student *t*-tests. Data were considered statistically significant with *p* < 0.05.

## 5. Conclusions

The current study demonstrates that CBD and HU308 decrease the number of 100K EVs released from HIV-1-infected monocytes. Furthermore, it shows that both cannabinoids decrease viral RNA TAR and *env* within the 100K subpopulation. Proinflammatory mRNA *IL*-1β and *TNF*-α was also shown to decrease with HU308 in the 2K (and to a lesser extent with CBD). This trend of decrease in viral RNA was seen in primary macrophages and 3D-neurospheres, and HU308 showed a decrease in the proinflammatory mRNA from infected neurospheres. Using an NSG humanized mouse model, mice showed a decrease of viral RNA with HU308 treatment. Taken together, these data indicate that HU308 or CBD decrease viral RNA, and that HU308 and, to some extent, CBD decrease proinflammatory cytokine mRNA released in EVs. Therefore, either CBD or HU308 could potentially be used in combination with cART to target both pro-inflammatory and viral gene expression for the prevention of HAND.

## Figures and Tables

**Table 1:** Summary of Results.

	EV Subpopulation	CBD	HU308
**EV Concentration**	**2K**	**##**	**###**
	**10K**	**###**	**-**
	**100K**	***	***
	**167K(S)**	**-**	**
	**167K(L)**	**###**	**-**
**Median Size**	**2K**	**-**	**-**
	**10K**	**-**	**-**
	**100K**	**-**	*
	**167K(S)**	**-**	**##**
	**167K(L)**	**	***
**Mean Size**	**2K**	**-**	**-**
	**10K**	**-**	*
	**100K**	**-**	*
	**167K(S)**	**-**	**##**
	**167K(L)**	**-**	**
**Peak Size**	**2K**	**-**	**-**
	**10K**	**-**	**-**
	**100K**	**-**	**-**
	**167K(S)**	**-**	**-**
	**167K(L)**	*	**
**TAR**	**2K**	**-**	**##**
	**10K**	**-**	**##**
	**100K**	***	**
	**167K(S)**	**	*
	**167K(L)**	**#**	**-**
***env***	**2K**	**-**	**-**
	**10K**	**-**	**-**
	**100K**	**	**
	**167K(S)**	**-**	**-**
	**167K(L)**	**#**	*
***TNF*-α**	**2K**	*	***
	**10K**	**#**	**-**
	**100K**	**-**	**-**
	**167K(S)**	**-**	**-**
	**167K(L)**	**#**	**-**
***IL*-1β**	**2K**	**-**	***
	**10K**	**#**	**##**
	**100K**	**-**	**-**
	**167K(S)**	**#**	**-**
	**167K(L)**	**#**	**-**
**Primary Macrophage** **+ cART (low)**	**TAR**	***	***
	**TAR-*gag***	***	**-**
	***env***	***	***
**Primary Macrophage**	**TAR**	***	***
	**TAR-*gag***	**	**
	***env***	***	***
**Neurosphere**	**TAR**	***	***
	**TAR-*gag***	**	***
	***env***	*	***
	***IL*-1β**	**###**	*
	***TNF*-α**	**###**	*
**Mouse**	**HU308 + cART (low)**	**TAR**	**
		**TAR-*gag***	*
		***env***	**
	**HU308**	**TAR**	**
		**TAR-*gag***	*
		***env***	*

Summary of results depicting significance, where “*” indicates a decrease, and “#” indicates an increase compared to the control; “*/#” = *p* < 0.05, “**/##” = *p* < 0.01, and “***/###” = *p* < 0.001.

## Data Availability

Data are contained within the article and the Supplementary Materials.

## Supplementary Materials

The following supporting information can be downloaded at: https://www.mdpi.com/article/10.3390/ph16081147/s1, Figure S1: Levels of viral and autophagy protein were assessed from EVs secreted from HIV-1-infected cells; Figure S2: Levels of proteins important to EV biogenesis and autophagy were assessed from EVs secreted from HIV-1-infected cells; Figure S3: Cell viability analysis of CBD and HU308 on monocytes and T-cells; Figure S4: Levels of HTLV-1 viral and proinflammatory RNA were assessed from neurospheres.
