# Supplementary material for: The Use of CBD and Its Synthetic Analog HU308 in HIV-1-Infected Myeloid Cells
Source: Pharmaceuticals (Basel). 2023 Aug 12;16(8):1147. doi: 10.3390/ph16081147 (PMC10458222; doi:10.3390/ph16081147)
Supplement: Supplementary file 1 [file pharmaceuticals-16-01147-s001.zip › Williams et al. 2023 Supplementary Figures.pdf]

Supplementary Materials

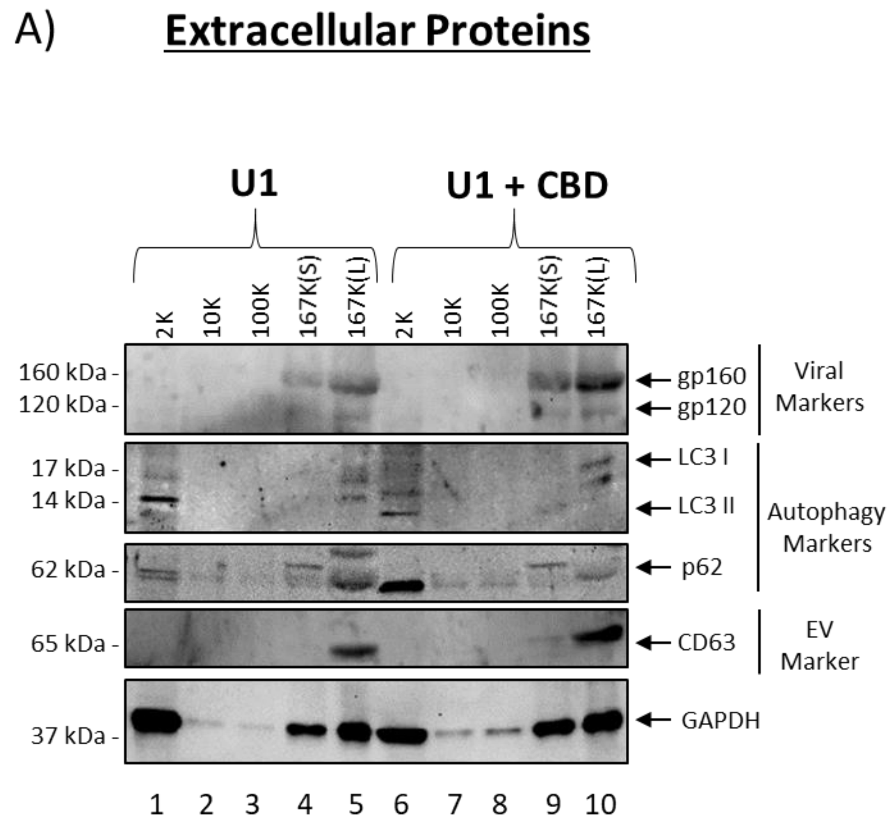

Sup. Figure S1. Levels of viral and autophagy protein were assessed from EVs secreted from HIV-1-infected cells. U1 cells (1 x 10^6 cells/mL) were treated with or without CBD (5 μM) for 3 days. At 72 hr cell pellets were collected and EV subpopulations were isolated (2K, 10K, 100K, 167K(S), and 167K(L)). EV subpopulations were assessed for HIV-1 envelope protein mature form (gp120) and unprocessed form (gp160), autophagy markers LC3 I and LC3 II, and p62, as well as EV marker CD63.

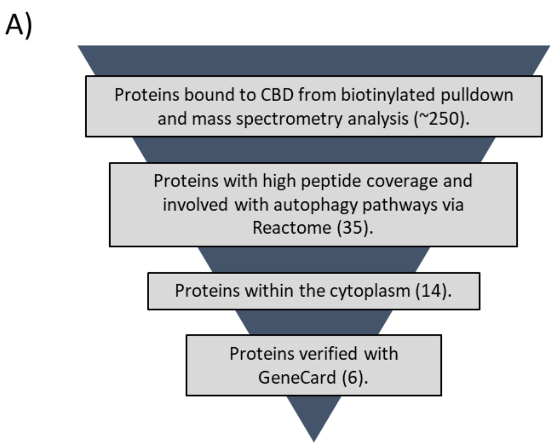

B)

Protein	Autophagy	Classical Exosome Biogenesis
Catalase	+	-
Hsc70	+	+
HSP90	+	-
Cofilin	+	-
Coronin 1A	+	+
Rab7	+	+

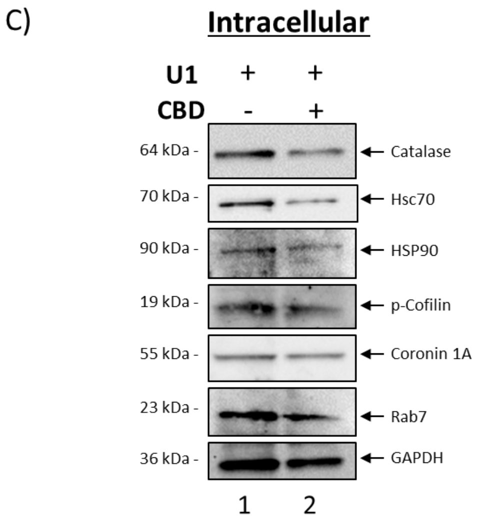

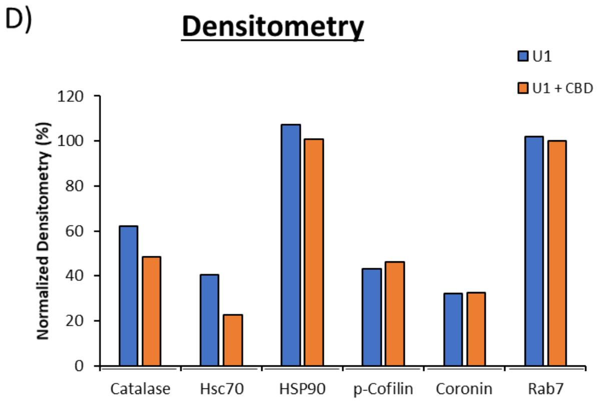

Sup. Figure S2. Levels of proteins important to EV biogenesis and autophagy were assessed from EVs secreted from HIV-1-infected cells. Workflow of protein selection from biotinylated pull down to verification with gene card (A) (MS data available in Supplementary data from DeMarino et al. 2022). Description of selected proteins and role in autophagy and classical exosome biogenesis (B). U1 cells (1 x 10^6 cells/mL) were treated with or without CBD (5 μM) for 3 days and the cell pellets were collected, lysed and were processed for Western Blot. The membrane was imaged for Catalase, Hsc70, HSP90, p-Cofilin, Coronin 1A, and Rab7 (C), these were then normalized to GAPDH via ImageJ densitometry (D).

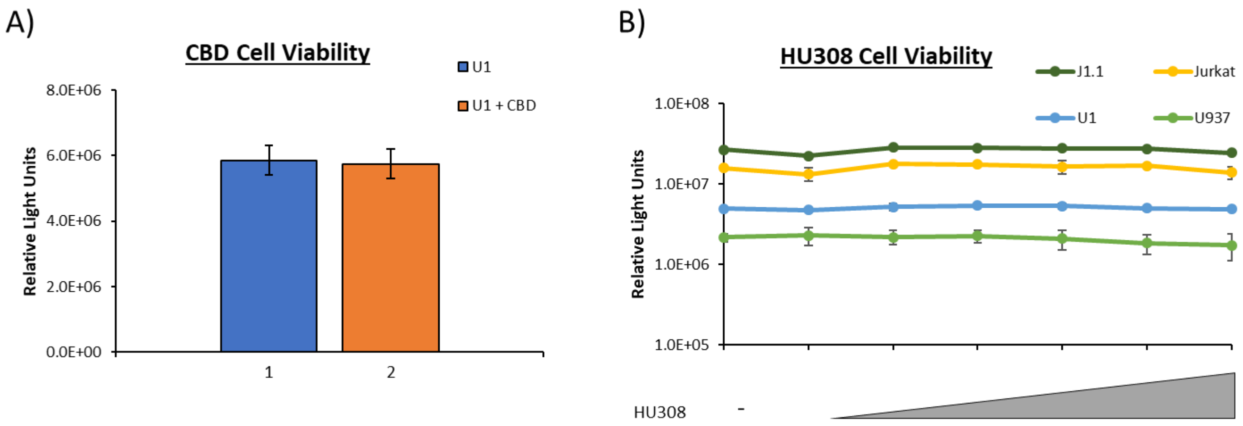

Sup. Figure S3. Cell viability analysis of CBD and HU308 on monocytes and T-cells. HIV-1-infected U1 cells (5 x 10^4) were treated with CBD (5 µM) daily for 3 days (A). Uninfected and HIV-1 infected monocytes (U937 and U1 respectively; 5 x 10^4) and uninfected and infected T-cells (Jurkat and J1.1; 5 x 10^4) treated with HU308 at (0.1, 1, 5, 10, 100 µM) daily for 3 days (B). Cell viability was determined using Cell Titer-Glo assay at day 3. Student's t-test compared treated samples to untreated samples. *, $p < 0.05$; **, $p < 0.01$; ***, $p < 0.001$. Error bars, S.D.

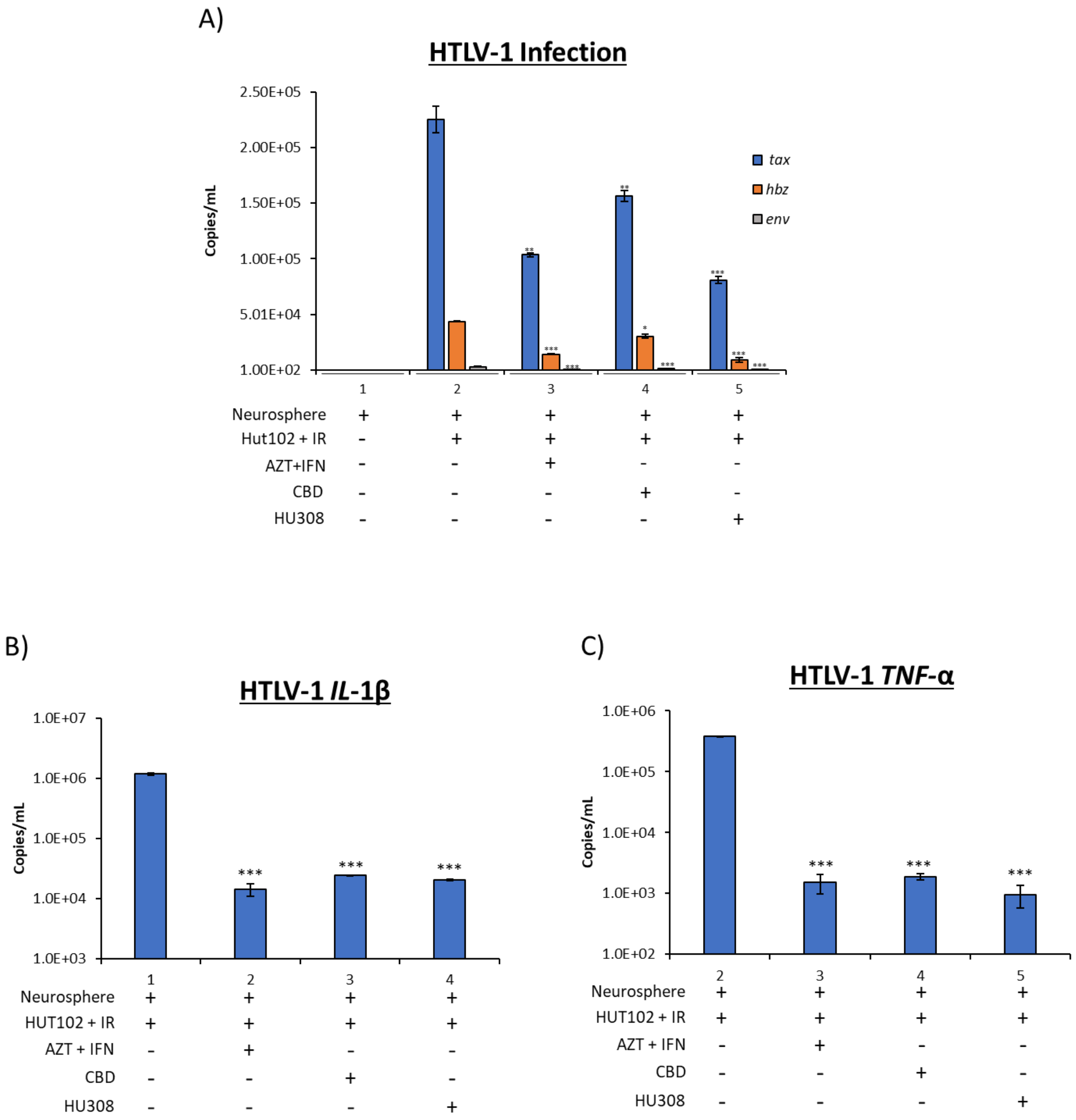

Sup. Figure S4. Levels of HTLV-1 viral and proinflammatory RNA were assessed from neurospheres. Neurospheres were co-cultured with HTLV-1 as described in Branscome et al. 2022. Briefly, HUT102 cells were irradiated and then cultured with differentiated neurospheres (1:100 approximate ratio of infected cells: uninfected cells) with or without IFN (10 K unit), AZT (20 µM), CBD (10 µM), or HU308 (10 µM). Cultures were treated every 2 to 3 days for 14 days. RNA was isolated and assessed for levels of viral *tax*, *hbz*, and *env* (A), as well as *IL*-1β (B) and *TNF-α* (C). Student's t-test compared treated samples to untreated samples. *, $p < 0.05$; **, $p < 0.01$; ***, $p < 0.001$. Error bars, S.D.
